# OX26-cojugated gangliosilated liposomes to improve the post-ischemic therapeutic effect of CDP-choline

**DOI:** 10.1007/s13346-024-01556-3

**Published:** 2024-03-13

**Authors:** Nicola d’Avanzo, Donatella Paolino, Antonella Barone, Luigi Ciriolo, Antonia Mancuso, Maria Chiara Christiano, Anna Maria Tolomeo, Christian Celia, Xiaoyong Deng, Massimo Fresta

**Affiliations:** 1 Department of Clinical and Experimental Medicine, University of Catanzaro “Magna Graecia”, Viale “S. Venuta”, 88100 Catanzaro, Italy; 2 Department of Health Sciences, University of Catanzaro “Magna Graecia”, Viale “S. Venuta”, 88100 Catanzaro, Italy; 3 Department of Medical and Surgical Sciences, University of Catanzaro “Magna Graecia”, Viale “S. Venuta”, 88100 Catanzaro, Italy; 4 Department of Cardiac, Thoracic and Vascular Science and Public Health, University of Padova, 35128 Padua, Italy; 5 Perdiatric Research Institute “Città della Speranza”, Corso Stati Uniti, 4, 35127 Padua, Italy; 6 Department of Pharmacy, University of Chieti – Pescara “G. d’Annunzio”, Via dei Vestini 31, 66100 Chieti, Italy; 7 Lithuanian University of Health Sciences, Laboratory of Drug Targets Histopathology, Institute of Cardiology, A. Mickeviciaus g. 9, LT-44307 Kaunas, Lithuania; 8 Institute of Nanochemistry and Nanobiology, School of Environmental and Chemical Engineering, Shanghai University, Shanghai, 200444 China

**Keywords:** Liposomes, Brain targeting, Nanomedicine, CDP-choline, Stroke

## Abstract

Cerebrovascular impairment represents one of the main causes of death worldwide with a mortality rate of 5.5 million per year. The disability of 50% of surviving patients has high social impacts and costs in long period treatment for national healthcare systems. For these reasons, the efficacious clinical treatment of patients, with brain ischemic stroke, remains a medical need. To this aim, a liposome nanomedicine, with monosialic ganglioside type 1 (GM1), OX26 (an anti-transferrin receptor antibody), and CDP-choline (a neurotrophic drug) (CDP-choline/OX26Lip) was prepared. CDP-choline/OX26Lip were prepared by a freeze and thaw method and then extruded through polycarbonate filters, to have narrow size distributed liposomes of ~80 nm. CDP-choline/OX26Lip were stable in human serum, they had suitable pharmacokinetic properties, and 30.0 ± 4.2% of the injected drug was still present in the blood stream 12 h after its systemic injection. The post-ischemic therapeutic effect of CDP-choline/OX26Lip is higher than CDP-choline/Lip, thus showing a significantly high survival rate of the re-perfused post-ischemic rats, i.e. 96% and 78% after 8 days. The treatment with CDP-choline/OX26Lip significantly decreased the peroxidation rate of ~5-times compared to CDP-choline/Lip; and the resulting conjugated dienes, that was 13.9 ± 1.1 mmol/mg proteins for CDP-choline/Lip and 3.1 ± 0.8 for CDP-choline/OX26Lip. OX26 increased the accumulation of GM1-liposomes in the brain tissues and thus the efficacious of CDP-choline. Therefore, this nanomedicine may represent a strategy for the reassessment of CDP-choline to treat post-ischemic events caused by brain stroke, and respond to a significant clinical need.

**Graphical abstract:**

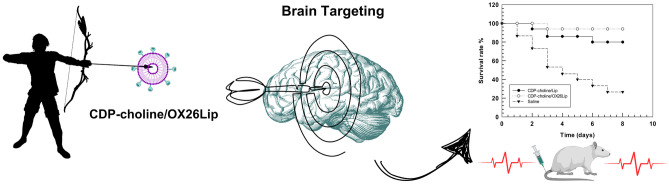

**Supplementary Information:**

The online version contains supplementary material available at 10.1007/s13346-024-01556-3.

## Introduction

Cerebrovascular stroke is currently one of the major causes of death worldwide, with a mortality rate of 5.5 million per year or long-term adult disability in 50% of survived patients [1, 2], as well as high comorbidity healthcare costs [3–5].

Clinical studies and case reports showed an increasing incidence of cerebral stroke because of several risk factors [2], as well as the socioeconomic improvement, business development, and environmental pollutants [6]. All these components increase hypertension and hypercholesterolemia, diabetes, and obesity in persons that had a fatty diet [6].

Stroke is a complex disease which includes ischemic and hemorrhagic events, caused by the partial or total obstruction of blood vessels and a significant reduction of the oxygen flux and glucose uptake in specific brain’s areas, or the bleeding in the brain tissue, with the following collapse of capillary vessels [7, 8].

To date, ischemic stroke is the most prevalent form of stroke worldwide and it is characterized by a primary damage in the brain ischemic core and a rapid secondary damage in the neighbor areas, or penumbra zone [2, 9]. The extent of neuronal damage is strongly affected by timing occurred between ischemia and reperfusion process [10]. The delay in the primary restoring procedure of ischemic stroke leads to the impairment of the different surrounding brain tissues’ function, thus eliciting a significant modifications of biochemical and metabolic pathways, with the final result of a post-ischemic neurodegeneration [11]. Although no therapeutic strategies are currently available to reduce damages in the ischemic core, different therapies are used to treat post-ischemic maturation process and to reduce injuries associated with brain neighboring area [12].

In this scenario, the brain supplements play a crucial role for restoring the damaged-no-death neurons and in particular, CDP-choline was a blockbuster drug which has been widely used to reduce the stroke-associated injuries [13, 14]. Despite clinical randomized trial in 2012 showed no significant therapeutic activity of CDP-choline in patients following ischemic stroke [15, 16], several preclinical studies in ischemic animal models were carried out, and demonstrated that the lack of CDP-choline effectiveness was due to its poor pharmacokinetic and physicochemical properties, which strongly limit the CDP-choline transport across the blood brain barrier (BBB) [17].

Nanotechnology, and the use of drug delivery systems, may overcome these drawbacks and improves both the targeting of injured tissues as well as the biopharmaceutical properties of CDP-choline after systemic administration. Liposomes significantly improved the therapeutic effect of CDP-choline in ischemic rats [18–21]. This improvement and the relative safety profile of CDP-choline delivery strongly supported the hypothesis of lipid-based nanomedicines for future clinical trials [17].

The brain targeting nanomedicines provided several options to increase the uptake and accumulation of payloads in different brain areas and had a selective efficaciousness with decreased systemic side effects [22, 23]. Biomarkers and mediator receptors, overexpressed in the brain capillary endothelial cells of BBB and involved in basal and metabolic brain physiological functions, provide a huge platform for brain targeting of nanomedicines after local or systemic injections [24].

The anti-transferrin receptor antibody conjugated to liposomes increased intracellular uptake and tissues targeting [25–27]. Recently, the gangliosides have been reconsidered as potential neuroprotective agents to treat brain degenerative diseases [28, 29]. The great interest of the scientific community is further demonstrated by the clinical trial (phase I) of Talineuren, a monosialic ganglioside type 1 (GM1)-based liposomes for the treatment of Parkinson disease. Moreover, monosialic gangliosides decreased the potential immunogenicity of polymers, like PEG that are used for the long-circulation of nanocarriers after systemic injection [30], and may represent a valid option for the development of nanomedicine-based therapies for brain disorders.

The aim of this work is the preparation of CDP-choline-loaded GM1-liposomes conjugated with anti-transferrin receptor antibody (OX26) for brain targeting delivery of this drug, and the improve of CDP-choline therapeutic effect in the brain ischemic stroke. OX26-conjugated CDP-choline-loaded GM1-liposomes were prepared, physicochemical characterized and tested in vivo on ischemic stroke rat models. Results demonstrated that OX26-bearing liposomes have suitable pharmacokinetic and biopharmaceutical properties for in vivo administration and increase the survival rate of ischemic and reperfusion rats.

## Materials and methods

### Materials

Cholesterol (CHOL), Sephadex G-25, phosphate buffered saline (PBS) solution and CDP-choline sodium salt hydrate were obtained from Merck (Milan, Italy). 1,2- dipalmitoyl-sn-glycero-3-phospocholine (DPPC), 1,2-dipalmitoyl-sn-glycero-3-phospho-L-serine (DPPS); 1,2-distearoyl-sn-glycero-3-phosphoethanolamine-N-[maleimide (polyethylene glycol)-2000] (DSPE-PEG2000mal), N-(carbonyl-methoxypolyethylene glycol-2000)-1,2-distearoyl-sn-glycero-3 phosphoethanolamine (DSPE-mPEG2k) and Monosialo Ganglioside (GM1) were purchased from Avanti Polar (Merck, Milan, Italy). [^3^H]-cholesteryl hexadecyl ether ([^3^H]CHE, 40 Ci/mmol) was purchased from Perkin Elmer-Italia (Monza, Italy). Mouse monoclonal transferrin receptor antibody (OX26) was obtained from BD Biosciences (Milan, Italy).

All the other reagents, that used during the experiments, were of analytical grade and are used without any further purification. Wistar rats were obtained from Harlam (Italy s.r.l. San Pietro al Natisone (UD), Italy).

## Methods

### Liposome preparation

Liposomes were prepared by using thin layer evaporation method with some modifications as previously reported [19, 31]. Briefly, DPPC, DPPS, Chol, GM1 and DSPEmPEG2000-mal, final molar ratio of 3:3:3:0.8:0.2, were co-dissolved by using an organic solvent mixture (chloroform: methanol, 3:1 v/v) in a round glass vial. The organic solvent was removed by using a rotavapor Büchi R-210 at 45 °C (Büchi, Milan, Italy) connected to a vacuum pump. The thin lipid film was hydrated with an aqueous solution of 40 mg/mL of CDP-choline (PBS 10 mM, pH 6.8), and a final lipid concentration of 50 mg/mL was obtained. Three minutes of warming at 60 °C and three minutes of vigorous stirring at 800 rpm were carried out for three times. The resulting multilamellar liposomes were warmed at 60 °C for 1 h and then frozen and thawed (5 min in liquid nitrogen and 15 min at 60 °C) ten-folds to improve the entrapment efficiency of the payload. The liposomal suspension was then extruded through polycarbonate filters with a pore size from 800 to 50 nm (Whatman^®^ Nuclepore^™^ Track-Etched Membranes, Merk Life Science S.r.l., Milan, Italy), by using a stainless-steel extrusion device (Lipex Biomembranes, Northern Lipids Inc., Vancouver, BC, Canada). The un-entrapped drug was removed by using Amicon^®^ Ultra centrifugal filters (cut-off 50 kDa).

The OX26 antibody was finally conjugated to the surface of small unilamellar liposomes as following reported.

Untargeted CDP-choline-loaded liposomes (CDP-choline/Lip) were prepared by replacing DSPEmPEG2000-mal with the same molar ratio of DSPEmPEG2000 during the lipid film preparation, while empty liposomes were made up by hydrating lipid film with PBS (pH 7.4, 10 mM). Radiolabeled liposomes were prepared by using [^3^H]-cholesteryl hexadecyl ether ([^3^H]CHE, 0.003% w/w) at a final concentration of 0.5 µCi/mL.

### Antibody conjugation

**Fig. 1:**
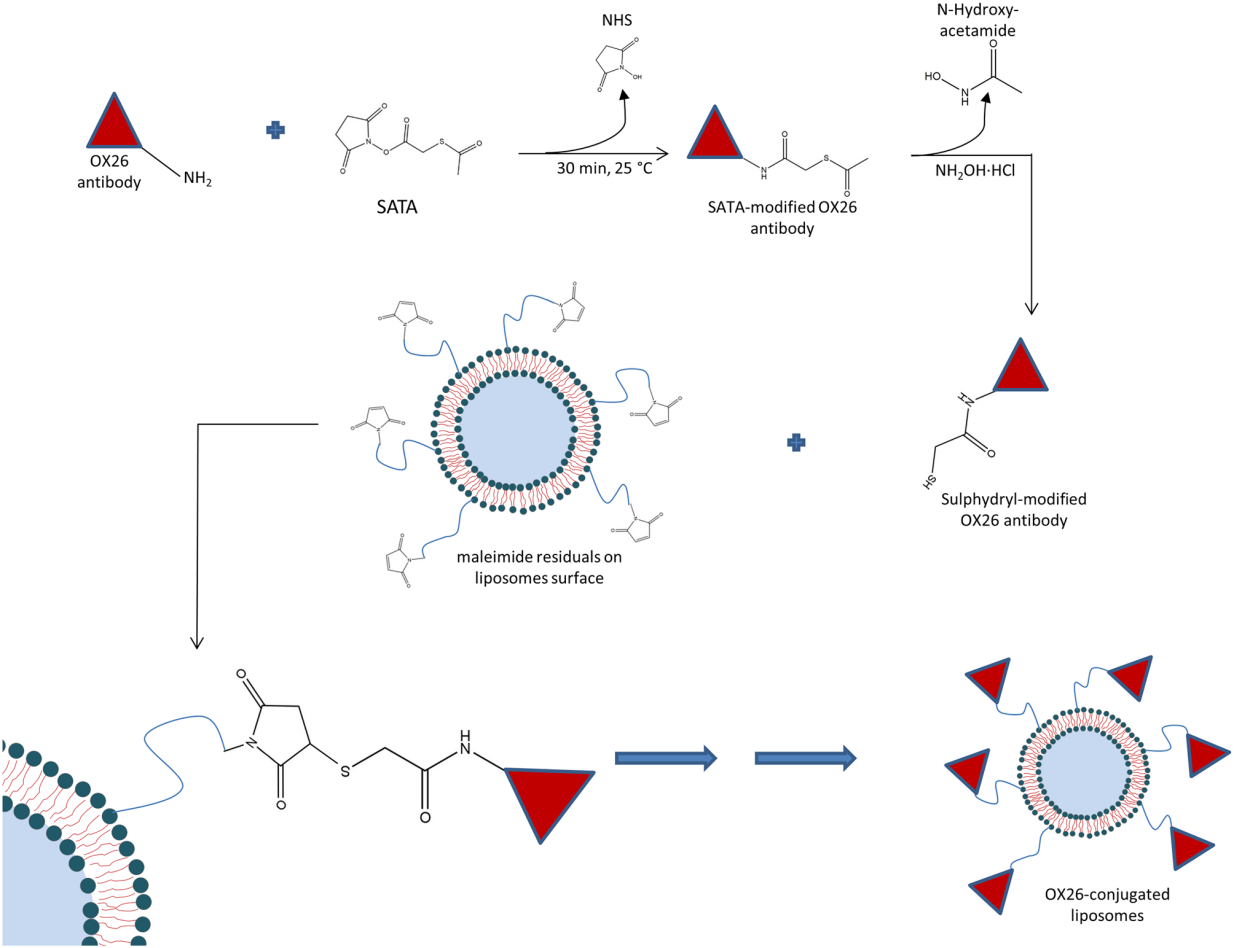
Schematic representation of OX26 conjugation on liposome surface. The conjugation of OX26 to liposomes was obtained between thiol group of OX26 and maleimide group of DSPE-PEG2000-mal

A sulfhydryl group was added to the N-terminal portion of OX26 antibody by using SATA reagent as previously reported elsewhere with some modifications [32]. A stock solution of SATA was prepared by dissolving this compound (6 mg) in 1 mL of DMF. 1 μL of resulting solution was added to 1 mL of OX26 solution (0.5 mg/mL in PBS at pH 7.4) to have a final molar ratio between SATA and antibody of at least 8:1. The resulting mixture was incubated for 30 min at room temperature and then purified by using amicon ultracentrifuge tube (cut-off 3 kDa, Merck Millipore S.A.S., France). The acetylated OX26 was stored at -80 °C upon their use, de-acetylated by incubation with de-acetylating solution (0.5 M hydroxylamine^.^HCl, 25 mM EDTA in PBS, pH 7.4) at room temperature for 2 h, and then conjugated to liposomes (Fig. 1). The thiolate antibody was purified with desalting columns pre-equilibrated with PBS buffer (PBS 10 mM, 10 mM EDTA, pH 6.8) and concentrated up to 1 mL by using amicon ultracentrifuge tube. The presence of thiol group on modified OX26 was carried out by the Ellman reaction (data not shown) as previously published [33].

Thiolate OX26 antibody (800 μL, 0.5 mg/mL) was conjugated to the surface of liposomes (lipid concentration 50 mg/mL) through the reaction between thiol group in the backbone of antibody and maleimide residual of DSPEmPEG2000-mal on the surface of liposomes by incubation at room temperature for 3 h, followed by an overnight incubation at 4 °C under continuous magnetic stirring (250 rpm) (Fig. 1). Ethanethiol was used at the end of reaction to block un-reacted maleimide residuals. The resulting OX26 liposomes were purified with exclusion chromatography by using the Akta Prime apparatus (GE Healthcare Bio-Sciences AB, Uppsala, Sweden) with a glass column packed with Sephadex G-25 and equipped with a spectrophotometer at fixed wavelength of 280 nm. When required, after purification, liposomes were concentrated by using total recovery amicon ultracentrifuge tube with a pore size of 50 kDa (Merck Millipore S.A.S., France).

### Physicochemical characterization

The average size, size distribution and zeta potential of liposomes were analyzed by using dynamic light scattering (DLS) technique as previously reported with some modifications [34]. Briefly, samples were diluted with isosmotic pyrogen free solution (1:50 v/v) to avoid multiscattering phenomena, and the analysis was carried out at 25 °C by using Zetasizer Nano ZS (Malvern Panalytical Ltd, UK). Results are the average of three independent analyses ± standard deviation (S.D.). The conjugated OX26 was indirectly quantified as previously reported with some modifications [35]. OX26-conjugated liposomes were centrifuged at 90,000 × g (1 h, 4 °C) and the concentration of OX26 in the supernatant was quantified by Mouse IgG2a ELISA quantification kit (Sigma Aldrich, Milan, Italy). The amount of OX26 conjugated on the liposome surface was then calculated by deleting the amount of unconjugated monoclonal antibody in the supernatant from total amount of OX26 that are used during the liposome preparation.

The morphology of nanovesicles was also studied by using TEM analysis, as previously published [36]. Samples were properly diluted in isosmotic buffer and dropped into coated grids. Uranyl acetate solution (2% w/v) was used to stain the samples after drying. TEM images were acquired by using a Veleta (Olympus Soft Imaging System) digital camera, operating at 100 kV with Tecnai G2 (FEI) transmission electron microscope (TEM).

### Drug entrapment efficiency and release kinetic of liposomes

The purified CDP-choline-loaded liposomes were dried under vacuum by using a ThermoScientific^™^ Savant^™^ SpeedVac^™^ (Fisher Scientific Italia, Rodano (MI), Italy) for 12 h. Dried liposomes were dissolved by using cooled methanol (+4 °C) and the CDP-choline entrapment efficiency was evaluated by using HPLC apparatus as previously reported by Lin et al. with some modification [37]. Samples were analyzed by using a C_18_ column (4.6 × 100 mm, 3 μm, Gemini-Nx plus C18; Phenomenex, CA, USA) at 25 °C. The mobile phase was methanol and KH_2_PO_4_ 0.5 M (10:90 v/v ratio, respectively) with a flow rate of 1 mL/min. CDP-choline detection was carried out by using a UV-detector connected to HPLC apparatus at 280 nm. Empty liposomes were used as blank and an external calibration curve of CDP-choline, in the range from 1 to 25 μg/mL, was used to quantify the drug inside liposomes. The drug loading percentage (D.L.%) (Eq. 1) and the entrapment efficiency percentage (E.E.%) (Eq. 2) were calculated by using the following equations:

1 $$D.L.\%=\frac{{D}_{en}}{{Lip}_{tot}}*100$$

2 $$E.E.\%=\frac{{D}_{en}}{{D}_{tot}}*100$$

where,* D*_*en*_ is the amount of drug loaded inside liposomes, while *Lip*_*tot*_ and D_tot_ are the total amount of lipid, and the total amount of drug that are used during the preparation procedure of liposomes.

The CDP-choline release from liposome was studied in vitro by using the bag dialysis method as previously published with some modification [38]. Briefly, liposomes were filled inside a cellulose acetate dialysis tube (Spectra/Por 1 Standard RC Dry Dialysis Tubing, 50 kDa, Spectrum Labs, USA) and hold in the receptor medium to have a final ratio between liposomes and receptor medium of 1:100 v/v. The release study was carried out at 37 ± 0.5 °C under a slow and continuous stirring (200 rpm) up to 24 h. At fixed time points (30 min, 1, 2, 3, 4, 6, 8, 10, 24 h), 1 mL of receptor medium was withdrawn and replaced with the same volume of fresh medium. Two different media, i.e. PBS (10 mM, pH 7.4) and PBS supplemented with 50% of human plasma, were used to study the kinetic release profile of CDP-choline. To avoid potential interference during the analysis, proteins of human plasma were removed. Briefly, a slight acid methanol solution was mixed with different samples (methanol/sample 3:1 v/v ratio), centrifuged at 11,000 × g for 10 min and the supernatant was then analyzed.

The percentage of CDP-choline released from liposomes was quantify by using the following equation (Eq. 3):

3 $$\mathrm{Drug \;released\; \%}=\left(\frac{{{\text{D}}}_{{\text{rel}}}}{{{\text{D}}}_{{\text{en}}}}*{\text{d}}.{\text{f}}.\right)\times 100$$

where, D_rel_ is the amount of drug released at specific time point, D_en_ is the amount of drug loaded inside liposomes and d.f. is the dilution factor between the volume of liposomes loaded in the dialysis tube and the volume of receptor medium. Any further dilutions, before the analysis, were considered to calculate the amount of CDP-choline released from liposomes. Empty liposomes were used as negative control.

### Stability in human plasma

The physical stability of liposomes was tested in a human plasma/PBS mixture (50:50 v/v ratio) (HP/PBS) as previously published with some modifications [39]. Briefly, 400 µL of CDP-choline-loaded OX26-conjugated liposomes (CDP-choline/OX26Lip) were incubated with 2 mL of HP/PBS medium (50% v/v) at 37 °C and then gently stirred up to 24 h. At fixed time points, 100 µL of the resulting mixture were analyzed by using DLS, and the average sizes of liposomes incubated with HP/PBS medium were measured. Liposomes incubated at the same conditions with saline solution (NaCl 0.9% w/v) were used as negative control during the experiment.

### Turbiscan lab expert analysis

Turbiscan Lab expert (Formulaction, L’Union, France) was used to test the long-term stability of liposomes at 37 and 25 °C as previously published [40]. Briefly, CDP-choline/Lip (control) and CDP-choline/OX26Lip were hold in a glass vial tube and diluted ten-times with PBS (10 mM, pH 7.4) up to a final volume of 6 mL. The analysis was carried out for the full height of samples (~ 10 mm) for 1 h. A pulsed infrared LED (wavelength of 880 nm) was used for different measurements and the results were reported as transmitted and backscattered lights through and by liposomes. Backscattering and transmittance were measured with optical detectors at 45° and 180°, for evaluating the long-term stability of liposomes. Potential sedimentation, creaming and/or flocculation of colloidal nanoparticles, like liposomes, did not occur with an instrument threshold below or equivalent to 5%. The results were reported for sample height ranging between 2.5 and 10 mm, because variations of backscattering and transmittance profiles over 5% at the sample height of 2 mm and/or over 10 mm are related to the presence of bubbles air at the bottom and top of glass holder, and they are not related to the occurrence of destabilization phenomena [40]. The global destabilization profiles (TSI) of liposomes were also recorded as a function of time up to 1 h of incubation. Moreover, the mean diameter of liposomes was also evaluated during the study and the potential variations have been reported as a function of time.

### Animals

Animal studies were carried in accordance with the Guide for the Care and Use of Laboratory Animals from directive 2010/63/EU of the European Parliament and protocols approved by the National Directorate of Veterinary Services (Italy, Permit No. 235 on June 30, 2011). Adult Wistar rats (250–300 g, body weight) were used for these studies and housed at 25 °C, 65% relative humidity, 12 h dark/12 h light cycle, with water and food ad libitum.

### Biodistribution studies

Long-circulating properties of CDP-choline/OX26Lip and the relative uptake in the main RES organs (i.e. liver and spleen) were studied by injecting [^3^H]-labeled liposomes in the tail vain of rat (average weight of ~ 270 g). At fixed time points after injection (3, 12 and 24 h) the animals were sacrificed, and the tissues were collected for the analysis. Three animals were used at different time points for each independent experiment, and three independent experiments were carried out. Briefly, organs were hold into polypropylene-based liquid scintillation cylinder vials (Sigma-Aldrich Chemie, GmbH, Steinheim, Germany) and incubated (4 h at 60 °C under continuous stirring) with 2 mL of quaternary ammonium hydroxide solution (Sigma-Aldrich Chemie, GmbH, Steinheim, Germany) to have a complete dissolution of tissues. Hydrogen peroxide (2 mL at 24% v/v) was used to decolorize the mixture and 7 mL of liquid scintillation cocktail (Ready Organic^™^, Beckman Coulter Inc., Fullerton, USA) was further added to samples and vigorously mixed. The resulting samples were quantified by using Wallac Win Spectral^™^ 1414 liquid scintillation counter coulter (PerkinElmer Life and Analytical Sciences, Inc. Waltham, MA, USA) and data were analyzed by 1414 Win Spectral Wallac LCS Software. The quantification of radio-labeled liposomes accumulated in different tissues was performed as previously published [41]. The signal intensity of endothelium and blood, which interfered with collected samples, was corrected and the following equation (Eq. 4) was used for the analysis:

4 $${R}_{tissue}={R}_{organ}-\left({V}_{0}* {C}_{t}\right)$$

where, R_tissue_ is the corrected radioactivity, R_organ_ is the level of radioactivity measured in the different samples, V_0_ is the total volume of interstitial fluid and vasculature calculated as a ratio between the whole organ radioactivity levels and the blood concentration 1 min after the injection of radio-labeled liposomes, and C_t_ is the blood concentration at time t. The radioactive intensity of organs collected from control (untreated rats) was used as a further correction factor.

### Induction of ischemic stroke in rats

The ischemic stroke in adult male Wistar rats (250–300 g) was induced according to experimental protocol previously reported with some modification [19]. Briefly, the animals were anesthetized [42] by isoflurane inhalation (2.5% in 100% oxygen) and then the ischemic stroke was induced by the bilateral occlusion of the common carotid arteries. 30 min after occlusion, the blood flow was restored, and the ischemic animals were split in different groups and injected with different formulations for the evaluation of the therapeutic activity.

### In vivo therapeutic activity

Therapeutic efficacy of CDP-choline/OX26Lip was carried out by injecting liposomes in Wistar rats, during the reperfusion process and once a day for six days. The survival rate of treated animals was studied up to 8 days after the induction of ischemic event. The survival rate percentage (%) was calculated according to the following equation (Eq. 5):

5 $$Survival \;rate\; \left(\%\right)=\frac{survived \;animals}{total \;animal \;treated}*100$$

Liposomes were intravenously injected into the tail vein at a CDP-choline dose of 20 mg/kg. CDP-choline/Lip were injected at the same drug dose. Saline solution was used as a control. For each group a total number of 15 animals were used (5 animals in each group for every independent experiment, with a total number of 3 independent experiments).

For the study of lipid peroxidation and lactate levels, 1 h after reperfusion, the animals were sacrificed and then the analysis was carried out as previously published [19].

### Statistical analysis

The statistical significance was carried out by One-way analysis of variance (ANOVA) and Tukey’s multiple comparison test. Analysis was performed by using SigmaPlot v.12 and Excel (Office 2010) and the significance levels was carried out for *p < 0.05, **p < 0.01 and ***p < 0.001.

## Results and discussion

### Physicochemical characterization

The optimization of physicochemical properties in drug delivery systems, such as particle size, size distribution, shape and interface properties, is one of the main challenge during the design of a potential nanomedicine, and it affects the biodistribution and metabolism of payloads as well as nanocarriers following the systemic injections [43]. Drug delivery systems can be further optimized for targeting tissue and physiological/pathological components. In these attempts, monosialic ganglioside (GM1) was used to make CDP-choline liposomes for two reasons: i) the intrinsic neuroprotective and antioxidant properties of GM1 [28, 29, 44, 45], that further increases the neurotrophic activity of CDP-choline; ii) the stealth properties of GM1 that has similar long-circulating properties of polyethylene glycol (PEG) but does not activate the anti-PEG immunogenic reactions [30].

The physicochemical characterization of CDP-choline/OX26Lip showed a mean size almost twice (84 ± 5 *vs* 54 ± 2, Table 1) compared to the untargeted CDP-choline/Lip, which has a lipid composition similar to those previously published by our research group [19]. The increase of nanoparticle size depended on the conjugation of OX26 on the liposomal surface. In fact, the hydrophilic property of the antibody increases the hydrodynamic diameter of liposome [46].

**Table 1:** Physicochemical properties

**Sample**	**Average diameter** **(nm)**	**Zeta-potential** **(mV)**	**PDI**
CDP-choline/Lip	54 ± 2	-36.6 ± 2.9	0.04 ± 0.01
CDP-choline/OX26Lip	84 ± 5***	-29.0 ± 2.3*	0.06 ± 0.03

The average diameter (nm), Z-potential and particle size distribution (PDI) were measured by using Zetasizer Nano ZS. Results are the average of three independent experiments ± standard deviation (S.D.)

Statistical significance *p < 0.05; **p < 0.01; ***p < 0.001

The conjugation of the OX26 antibody (final concentration 7.5 μg/μmol) further modified the Z-potential of liposomes from -36.6 ± 2.9 to -29.0 ± 2.3 (Table 1). In fact, the positively charged amino acids, present in the OX26 backbone, decreased the net negative surface charge of liposomes made up from DPPS and GM1 [31]. Conversely, the PDI values were below 0.1 and there were not any significant difference between CDP-choline/Lip and CDP-choline/OX26Lip (Table 1), thus showing a narrow size distribution of liposomes [47].

**Fig. 2:**
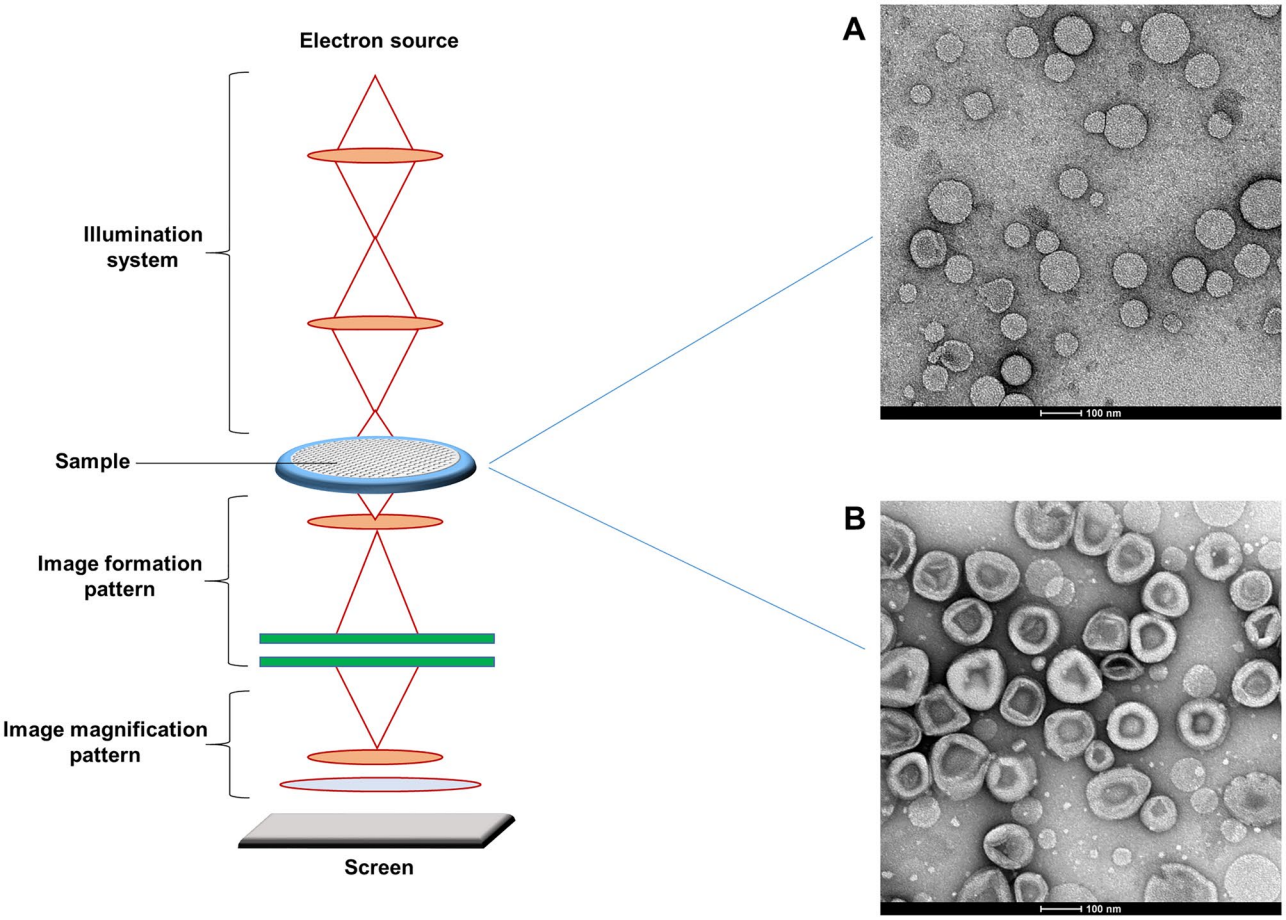
Schematic representation of transmission electron microscopy (left side) and representative images (right side). Panels **A** and **B** show CDP-choline/Lip and CDP-choline/OX26Lip. Scale bar: 100 nm. Images are representative of three independent analyses

DLS data agreed with TEM analysis (Fig. 2) and showed that liposomes had small unilamellar structures and a quite homogeneous size distribution. The TEM images showed that resulting liposomes had a round-shape morphology.

These data demonstrated that CDP-choline/OX26Lip have suitable physicochemical properties for in vivo administration and brain targeting, i.e. an average diameter below 100 nm [48], a net negative Z-potential value with suitable electrostatic repulsion between vesicles in suspension [49, 50], a narrow size distribution [51] and the presence of OX26 targeting molecules on the liposomal surface [35]. Namely, the conjugation of OX26 to liposomes, as well as their average sizes below the cut-off of brain vasculature fenestration during ischemic process, favors the liposome targeting into the brain after in vivo administration [18, 52].

### Entrapment efficiency and release kinetic of liposomes

**Fig. 3:**
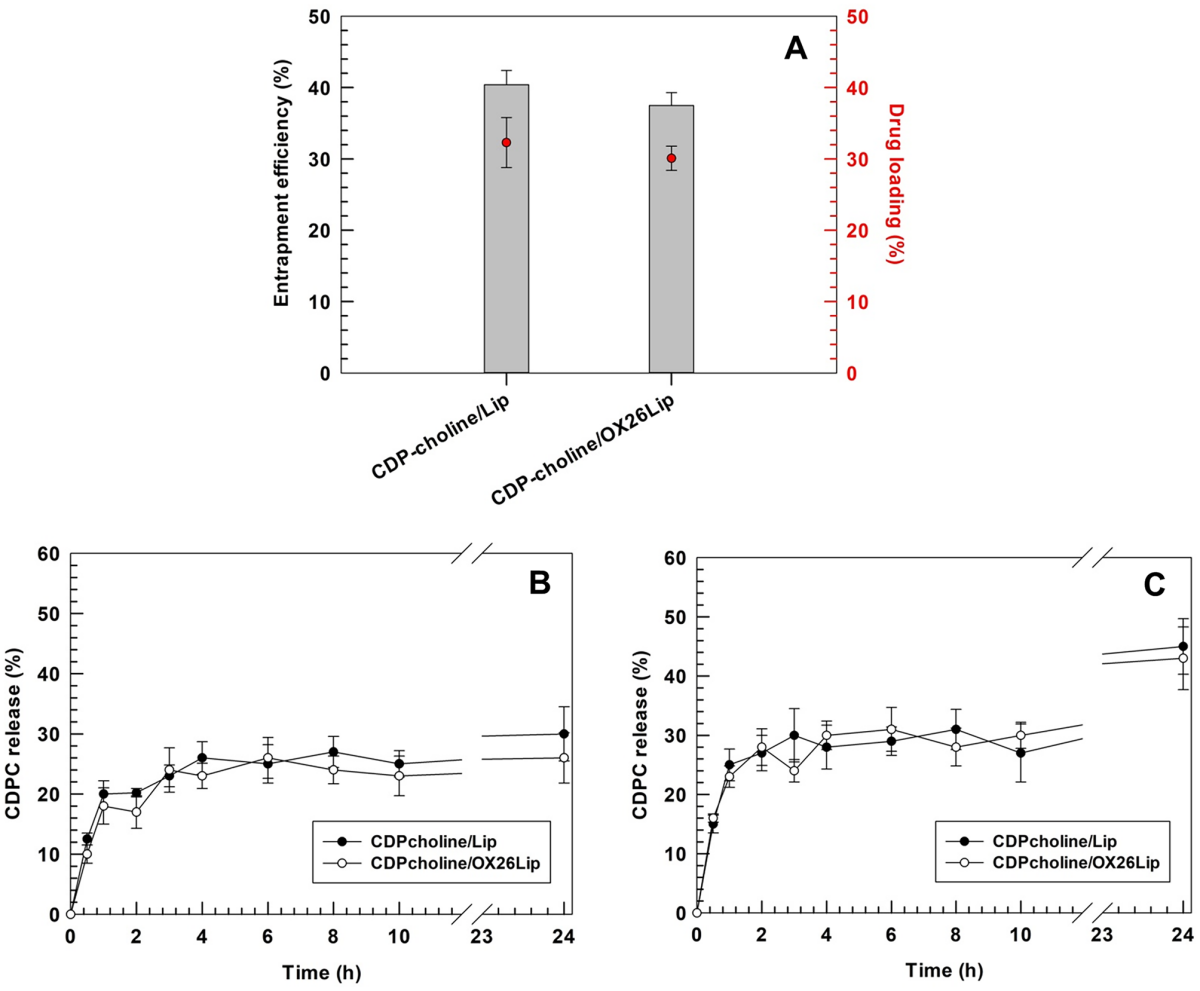
CDP-choline entrapment efficiency percentage, loading degree percentage and release kinetic profiles of liposomes. CDP-choline (drug) loading and entrapment efficiency percentage for conjugated (CDP-choline/OX26Lip) and unconjugated (CDP-choline/Lip) liposomes are reported in the panel **A**. The release kinetic of CDP-choline from liposomes was tested in vitro in PBS (panel **B**) and PBS supplemented with human plasma (PBS-HP) (50% v/v) (panel **C**), to simulate in vivo conditions after systemic injection. Results are the average of three independent experiments ± standard deviation (S.D.)

CDP-choline is loaded in the aqueous core of liposomes and size exclusion chromatography/ultrafiltration has been used to remove the un-entrapped drug and un-conjugated OX26, as reported elsewhere [53]. The presence of OX26 antibody on the surface of liposomes did not affect neither the loading efficiency nor the entrapment efficiency, 32.3 ± 3.5% and 40.4 ± 2.0% *vs* 30.1 ± 1.7% and 37.5 ± 1.8%, for CDP-choline/Lip and CDP-choline/OX26Lip, respectively (Fig. 3). Results agreed with data previously published by our research group, which had similar loading efficiency for CDP-choline in untargeted liposomes with similar lipid compositions [54].

CDP-choline/Lip and CDP-choline/OX26Lip showed a biphasic release profile with a rapid release of ~20% of CDP-choline during the first 2 h of incubation, followed by a pseudo-steady state up to 24 h if PBS was used as a receptor medium (Fig. 3B). Results obtained for the release kinetic profile of CDP-choline/Lip agreed data previously published by our research group [55], that studies liposomes with a similar lipid composition, and endorse that the presence of DSPEmPEG2000 at 2% molar ratio in the lipid bilayer did not modify the release of CDP-choline from liposomes. The rapid release of ~20% of CDP-choline may depend on the leakage of drug adsorbed on the external bilayer that makes hydrogen bonds with DPPS [18]. In fact, the larger volume of buffer in the receptor medium, than that loaded in the dialysis bag, generated a constant osmotic gradient that caused the leakage of ~20% CDP-choline.

Similar results were obtained for the release studies that have carried out in PBS-HP (50% v/v) up to 10 h of incubation (Fig. 3C). Conversely, a significant increase of drug release (15% higher than that obtained in PBS buffer) was obtained for both CDP-choline/Lip and CDP-choline/OX26Lip when the experiments were carried out in PBS-HP after 24 h of incubation (Fig. 3B, C). This difference may depend on the human plasma proteins dispersed in the receptor medium, which increase the osmotic pressure in the receptor medium and make some complexes with the released CDP-choline thus leading to an overall increased release of drug after 24 h of incubation. Moreover, the low molecular weight proteins, as well as the proteolytic fragment in the human plasma, can pass through the pores of polycarbonate dialysis tube, are adsorb on the surface of liposomes and modify the supramolecular structure and the interface properties of liposomes after 24 h of incubation. While, the ganglioside and PEG coated liposomes made a steric barrier on the liposomal surface that hampered the interaction between nanovesicles and human circulating proteins at early incubation times [56], thus resulting in a release kinetic profile that was similar to that obtained in PBS up to 10 h of incubation (Fig. 3B, C). The results showed that there is no significant variation of CDP-choline release between CDP-choline/Lip and CDP-choline/OX26Lip, thus highlighting that the antibody conjugation onto the nanovesicle surface did not affect the release of drug.

### Stability study of liposomes in human plasma

The interaction of nanomedicines with biological fluids can affect their stability, metabolism, biodistribution, targeting, and efficacy after systemic administration [57]. The circulating proteins, like albumin, can be adsorbed on the surface of liposomes, thus making protein corona that causes mechanical and chemical stresses on the nanovesicle surface and leads to the fast leakage of payloads [58, 59]. Protein corona also increases the rapid clearance of nanomedicines from blood circulation by macrophage uptake, and activates the immune systems [60, 61].

**Fig. 4:**
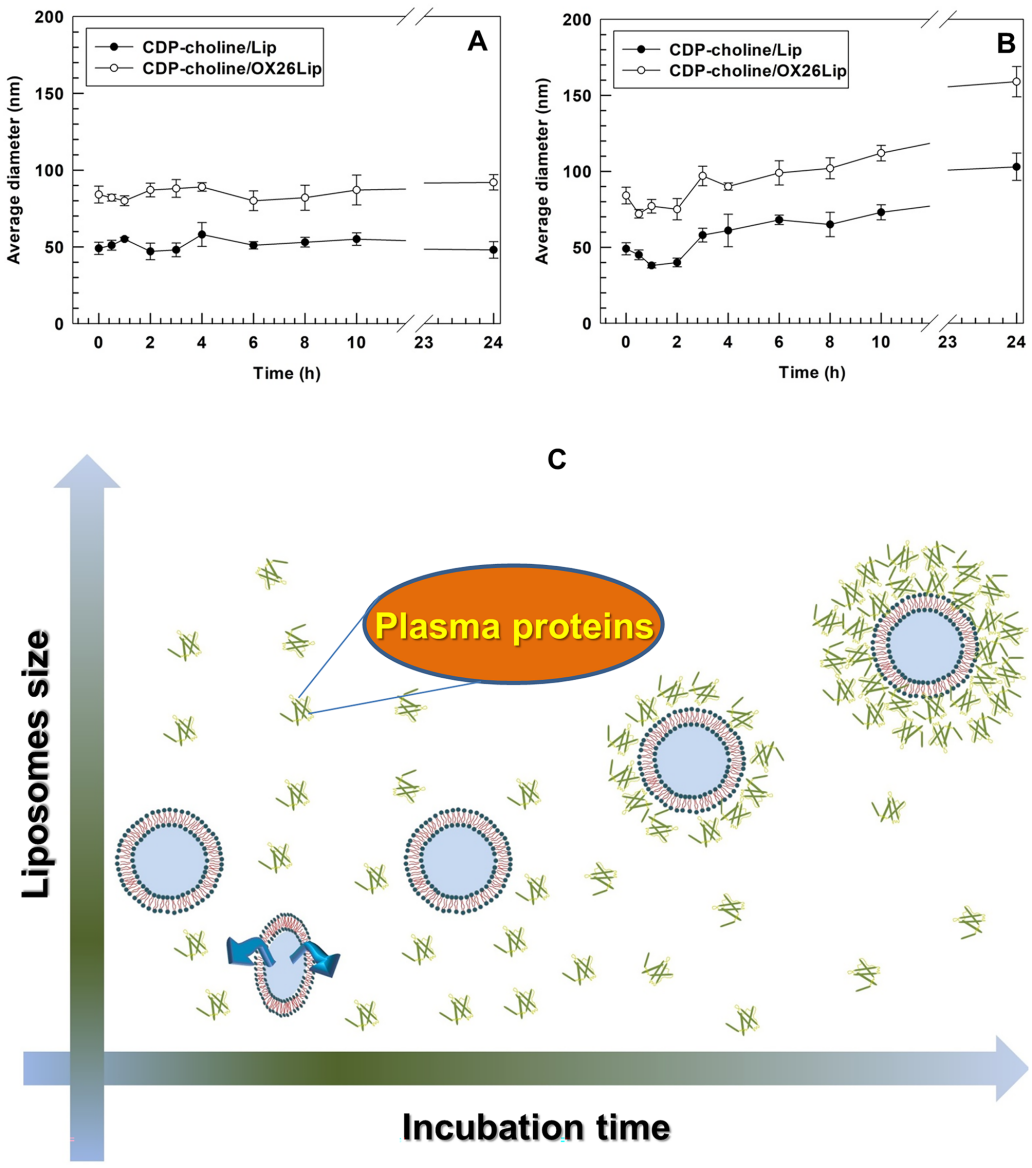
Mean size variation of CDP-choline/Lip and CDP-choline/OX26Lip in PBS buffer (**A**) and PBS supplemented with human plasma (50% v/v) (**B**). The analysis was carried out at 37 ± 0.5 °C. Results are the average of three independent experiments ± standard deviation (S.D.). PBS buffer (10 mM, pH 7.4) was used as a control during the experiment. *p < 0.05, **p < 0.01 and ***p < 0.001 were considered statistically significant. The statistical analysis has been reported in the Table S1. Panel **C** is a schematic representation of the interaction between liposomes and plasma proteins during incubation time

Biomacromolecules, such as ganglioside and PEG, preventing the opsonization process and limiting the activation of complement immune systems, can increase the physical stability of nanomedicines after systemic injection, and avoid the rapid leakage of payloads. In these attempts, we studied the stability of CDP-choline/Lip and CDP-choline/OX26Lip in PBS-HP by evaluating the average diameters of liposomes up to 24 h of incubation. A PBS solution (pH 7.4, 10 mM) was used as a control. The hydrodynamic diameters of CDP-choline/Lip and CDP-choline/OX26Lip were stable in PBS buffer up to 24 h of incubation (Fig. 4A).

Some variations of the CDP-choline/Lip and CDP-choline/OX26Lip for average sizes were obtained for nanovesicles incubated in PBS-HP (Fig. 4B). Namely, the average size of both nanomedicines slight decreased of ~10 nm after 2 h of incubation (Fig. 4B). The decrease of average sizes may depend on soft corona adsorbed on the liposomal surface, which caused a vesicle shrinkage due to the osmotic pressure generated on the external bilayer of the nanomedicines [62, 63]. The formation of a soft corona and the relative shrinkage of CDP-choline/Lip and CDP-choline/OX26Lip can further support the slight increase of CDP-choline release occurred after 2 h of incubation in PBS-HP (Fig. 3C).

By extending the incubation in PBS-HP up to 24 h, an increase in the CDP-choline/Lip and CDP-choline/OX26Lip mean size was obtained (Fig. 4B). This result can depend on the hard corona adsorbed on the surface of both nanomedicines. In fact, hard corona stuck the external bilayer of liposomes and changed their supramolecular structure [64, 65], thus leading to an increase of the nanomedicine hydrodynamic radius (Fig. 4B). The lack of nanomedicine aggregates, as evidenced by the absence of colloidal populations characterized by an average size equal to or greater than double the mean size of the nanomedicines before incubation, demonstrated that both CDP-choline/Lip and CDP-choline/OX26Lip were still stable following incubation in PBS-HP and no sedimentation occurred (Fig. 4B). This result was GM1- and PEG-dependent because both macromolecules shield the surface of both nanomedicines, thus hampering their aggregation after the interaction with circulating proteins [31].

### Long term stability studies

Turbiscan Lab analysis, which provided a non-invasive measurement of long-term stability of liposomes, like other colloidal nanoparticles [40, 66], were used to endorse the physical stability of CDP-choline/Lip and CDP-choline/OX26Lip. Turbiscan analysis can predict the long-term stability of colloidal systems by correlating the variation of backscattering (ΔBS) and transmission (ΔT) to destabilization phenomena, i.e. aggregation, flocculation, creaming and sedimentation [67, 68].

**Fig. 5:**
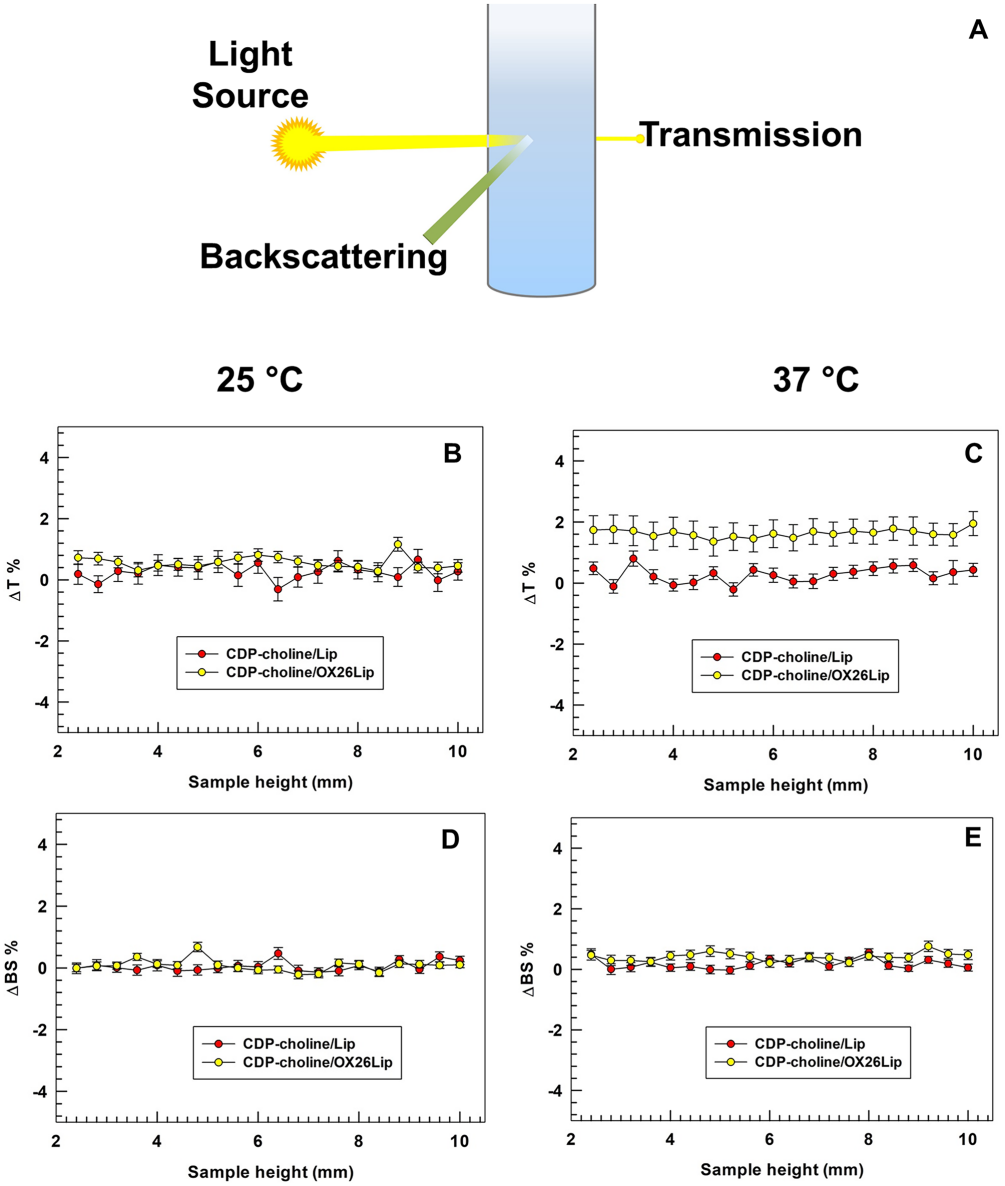
Variation of backscattering and transmission profiles of CDP-choline/Lip and CDP-choline/OX26Lip. Panel **A** is a schematic representation of Turbiscan analysis. The analysis was carried out at 25 °C (panels **B** and **D**) and at 37 °C (panels **C** and **E**). Results are representative of three independent experiments and are reported as a function of time (0-60 min) and sample height (mm)

The Turbiscan analysis was carried out both at 25 °C and 37 °C to simulate the room storage condition and body temperatures, respectively. ΔBS% and ΔT% was not over 5% for all tested samples (Fig. 5). These results demonstrated that CDP-choline/Lip and CDP-choline/OX26Lip are stable and there were no destabilization phenomena in agreement with previous published data [69].

**Fig. 6:**
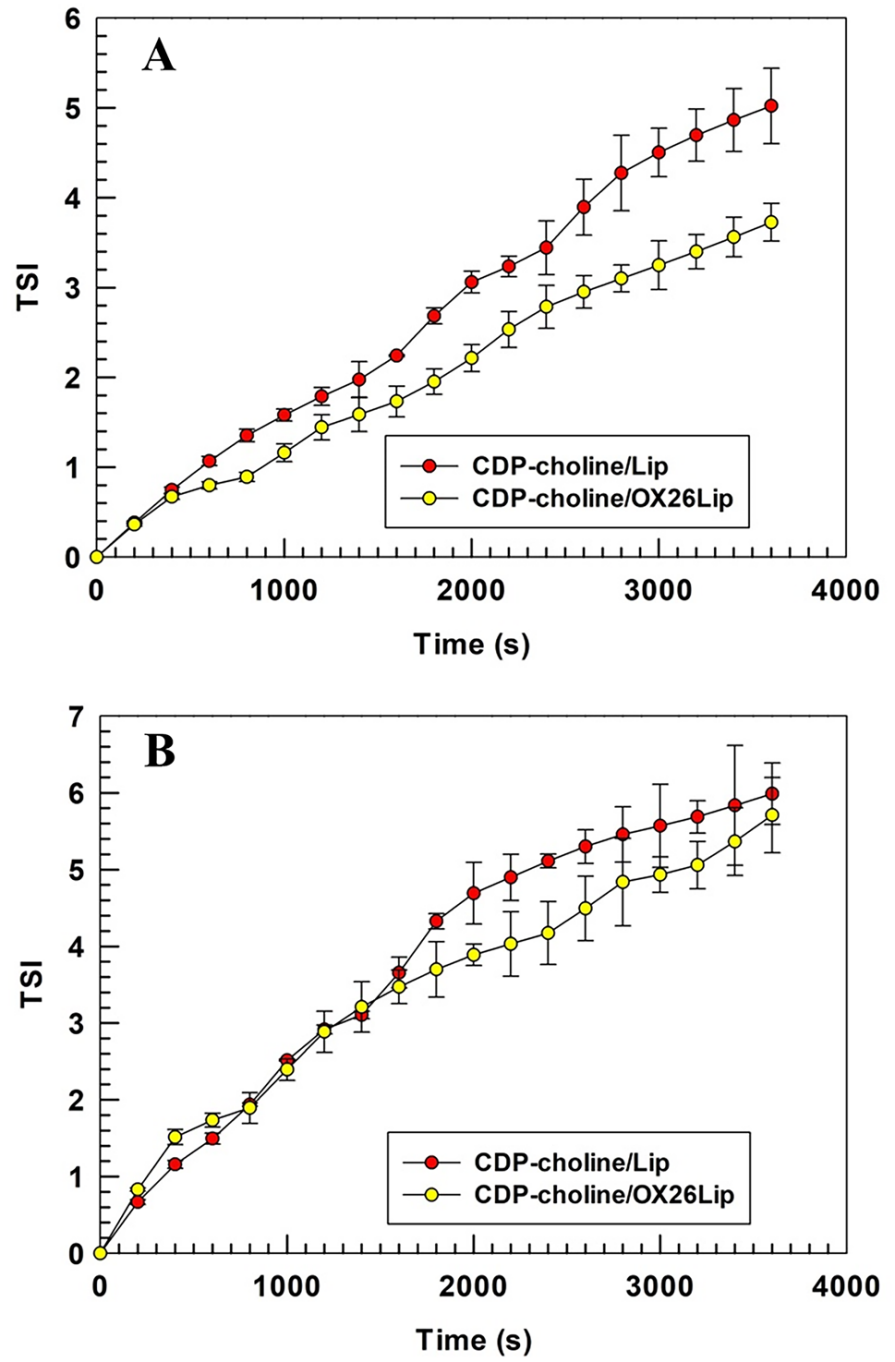
Turbiscan stability index (TSI) of CDP-choline/Lip and CDP-choline/OX26Lip. TSI was evaluated as a function of incubation time (0–1 h). The analysis was carried out at 25 ± 0.5 °C (**A**) and 37 ± 0.5 °C (**B**), and the results are representative of three independent experiments ± standard deviation (S.D.)

The absence of destabilization phenomena was further endorsed by the measurements of Turbiscan stability index (TSI), which corresponds to a TSI equal or below 6 for CDP-choline/Lip and CDP-choline/OX26Lip tested at 25 °C and 37 °C (Fig. 6). These results are in agreement with data previously published [34] and further highlighted the physical stability of CDP-choline/Lip and CDP-choline/OX26Lip according to ΔBS% and ΔT% data.

The colloidal stability of liposomes was also studied by analyzing their kinetic diameter profiles as a function of time for Turbiscan analysis. No significant variations were obtained during the incubation time, thus further endorsing the ΔBS%, ΔT% and TSI profiles as above reported (Fig. S1).

### Pharmacokinetic profiles and RES organs uptake

The biodistribution of nanomedicines, after systemic administration, affects their long-circulation and metabolism, as well as clearance. Enzymes and circulating proteins modify the biodistribution and long-circulation of nanomedicine because they change interface properties and polymer coating by different mechanisms [70, 71].

**Fig. 7:**
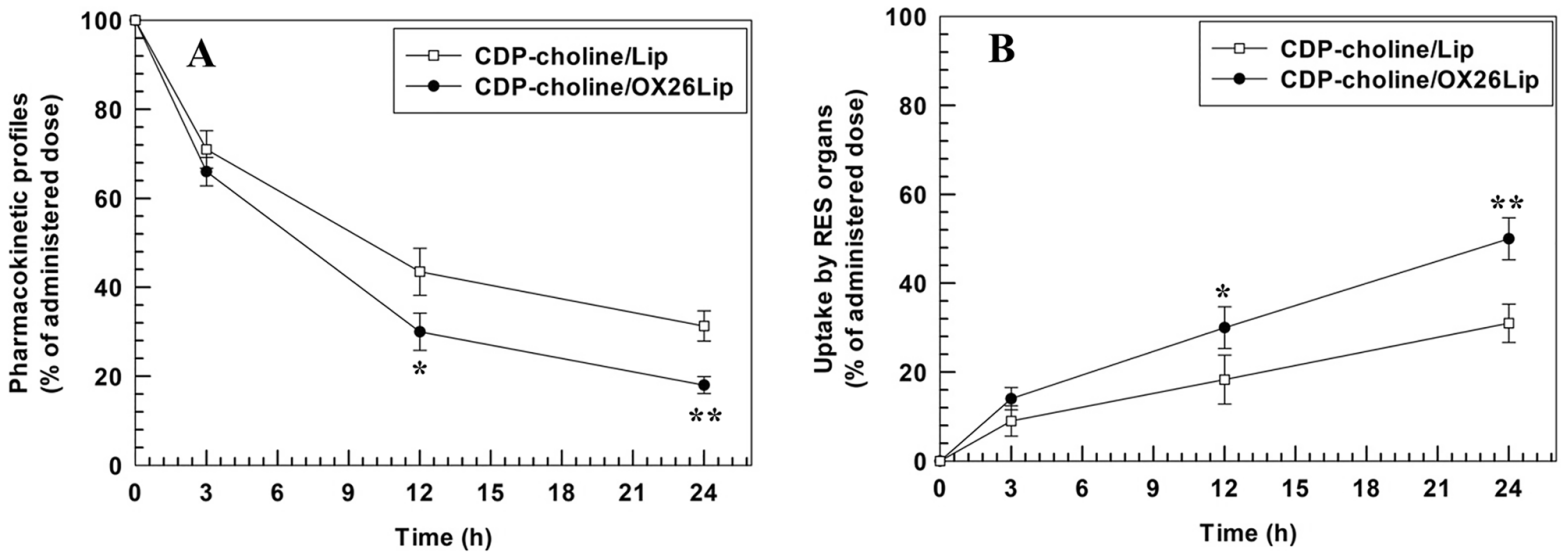
Pharmacokinetic profiles (**A**) and RES organs (liver and spleen) uptake (**B**) of CDP-choline/LipOX26 and CDP-choline/Lip. Results are the average of three independent experiments ± standard deviation (S.D.). Statistically significant: *p < 0.05, **p < 0.01

CDP-choline/LipOX26 had a lower blood circulation time than CDP-choline/Lip, and the results were in agreement with data previously published by our research group [55], The blood circulation time was 30.0 ± 4.2% (CDP-choline/LipOX26) and 43.5 ± 5.3% (CDP-choline/Lip) of the administered dose at 12 h after systemic injection (Fig. 7A). The amount of CDP-choline/LipOX26 in the blood decreased up to 18.3 ± 1.9% at 24 h after systemic injection, while the amount of CDP-choline/Lip in the blood was still 31.3 ± 3.4% (Fig. 7A). The lower blood circulation time of the CDP-choline/LipOX26 than the CDP-choline/Lip, having a similar lipid composition, may depend on the high accumulation of OX26-conjugated liposomes in the spleen (Fig. 7B), due to the overexpression of transferrin receptor into the spleen and liver [72]. Although these results may look like a disadvantage of CDP-choline/LipOX26 compared to CDP-choline/Lip, the transferrin receptors in the brain endothelial vessels promote the transport of liposomes across the BBB and allow their accumulation into the brain [73, 74]. Moreover, targeted nanomedicines bind their specific receptors immediately after systemic in vivo administration and at early circulations (where no significant difference were found in the pharmacokinetic profiles of unconjugated-liposomes and OX26-conjugated liposomes), because there is a minimum adsorption of circulating proteins on the surface of nanomedicines and protein corona did not stick on their surface [75, 76]. Our hypothesis further agreed data previously published that widely discussed the use of OX26 antibody as targeting moiety to accumulate nanocarriers inside the brain by transferrin receptor mediated uptake [35, 73, 77].

### In vivo therapeutic efficacy

The severity of brain damages, associated to ischemic event, depends on the timing of injected drug dosage between the stroke event and the brain reperfusion [78]. Indeed, the hypoxic conditions, related to ischemia, trigger several metabolic and electrolytic brain dysfunctions [79] as well as the release of various mediators, like the large production of reactive oxygen species (ROS), the lactate accumulation, the alteration of calcium homeostasis, the unbalance of potassium and sodium ions, the massive release of nitroxide. All these mediators supported the oxidative stress and provided the modification of macro environment [7, 80, 81], that caused the cell death during the ischemia [82–84]. In this attempt, a fast reperfusion of the tissues is needed to decrease the extension of the *penumbra zona*.

**Fig. 8:**
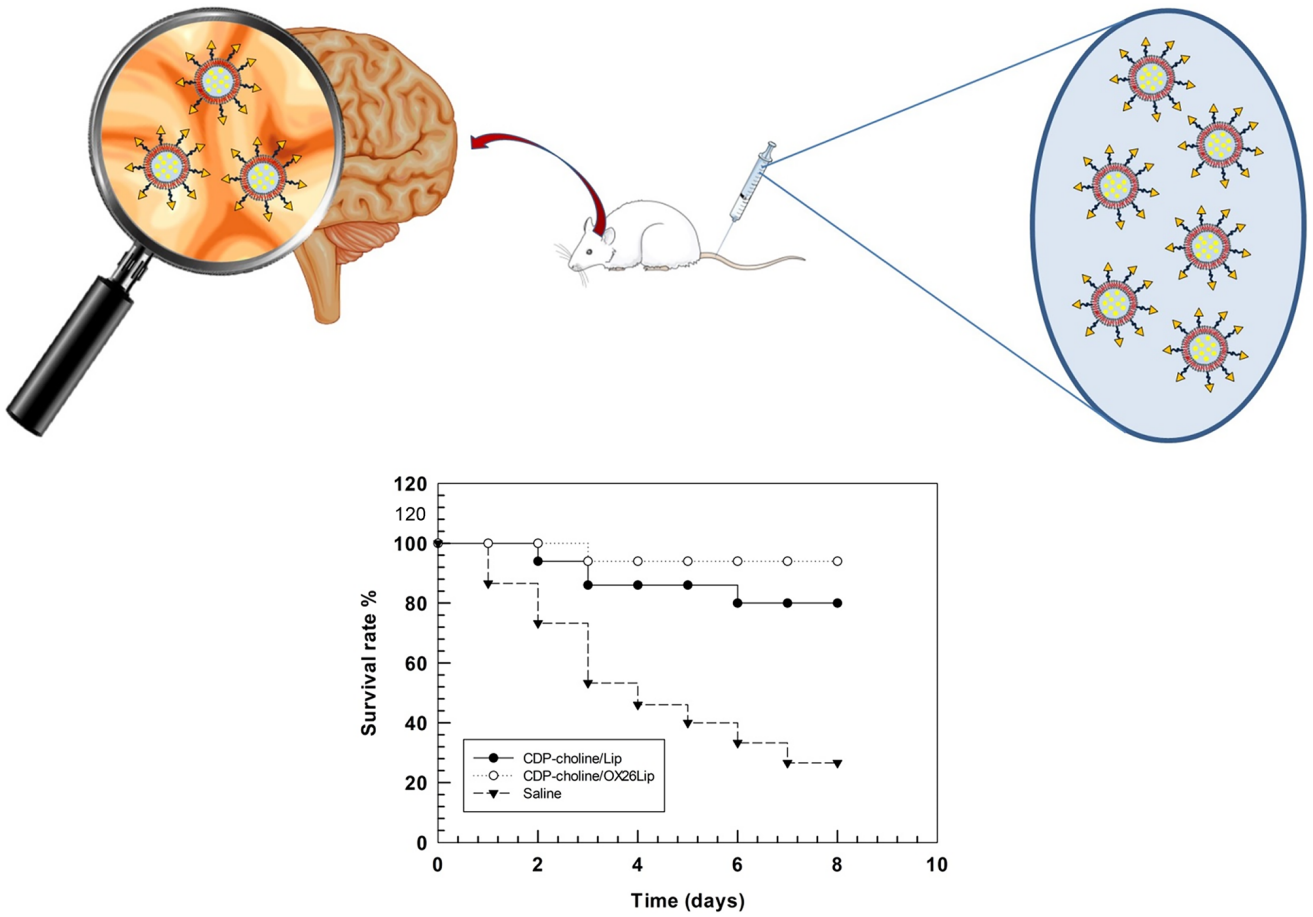
Survival rate of ischemic rat. The analysis was carried out after intravenous injection of CDP-choline/OX26Lip, CDP-choline/Lip or Saline solution 1 per day every day for 6 days (n = 15 for group). No significant variations of body weight were obtained for survived animals. Parts of the figure were drawn by using pictures from Servier Medical Art. Servier Medical Art by Servier is licensed under a Creative Commons Attribution 3.0 Unported License (https://creativecommons.org/licenses/by/3.0/)

We previously demonstrated that CDP-choline/Lip, with a similar lipid composition, significantly decreased the damage associated to ischemic stroke in rodents and improved the recovery of brain performance after ischemia [19, 54]. Although the reduced pharmacokinetic profiles of OX26-conjugated liposomes, the targeting strategy improved the accumulation of payload in the brain tissue and increased the survival rate of ischemic and re-perfusion rats from 78% to 96% after 8 days (Fig. 8). This improvement depended on the specific accumulation of CDP-choline in the brain mediated by liposomes.

The efficacy of CDP-choline/OX26Lip was also studied by analyzing the lactate and lipid peroxidation levels 1 h after the liposome (at the dose of 20 mg/Kg) administration during the reperfusion process. The improved efficacy of CDP-choline/OX26Lip was further demonstrated by the analysis of lipid peroxidation rate (Table 2) that measures the degenerative event catalyzed by the accumulation of radical species in the hypoxic area during ischemia followed by reperfusion process. The lipid peroxidation was evaluated as a function of conjugated dienes, and the resulting data demonstrated that the use of CDP-choline/OX26Lip significantly decreased the peroxidation rate of almost 5-times compared to CDP-choline/Lip (3.1 ± 0.8 *vs* 13.9 ± 1.1 mmol/mg proteins, respectively). Whereas no significant variation was obtained in the lactate accumulation in the rat that have been treated with CDP-choline/OX26Lip compared to those treated with CDP-choline/Lip (Table 2).

**Table 2:** Lactate and lipid peroxidation levels

**Sample**	**Lactate** (nmol/mg proteins)	**Diene peroxidation** (mmol/mg proteins)
CDP-choline/Lip	12.3 ± 0.7	13.9 ± 1.1(^###^)
CDP-choline/OX26Lip	10.8 ± 1.1	3.1 ± 0.8(^###^) (***)
Saline	10.6 ± 0.7	40.7 ± 6.3

The analysis was carried out 1 h after intravenous injection of CDP-choline/Lip, CDP-choline/OX26Lip or Saline solution after the re-perfusion of Wistar rats. Results are the average of five independent experiments ± standard deviation (S.D.)

Significance: ***p < 0.001 (CDP-choline/Lip *vs* CDP-choline/OX26Lip); ^###^*p* < 0.001 (liposomes *vs* saline)

The significant (***p < 0.001) decrease of lipid peroxidation rate demonstrated that the therapeutic efficacy of targeted CDP-choline/OX26Lip is higher than CDP-choline/Lip. These results demonstrated that CDP-choline/OX26Lip decreases the damage associated to the accumulation of ROS in the penumbra zone. This increase is higher than CDP-choline/Lip and caused a potentially reduction of post-ischemic neurodegeneration. CDP-choline/OX26Lip improved the accumulation of drug in the brain tissue and endorsed the higher targeting of CDP-choline/OX26Lip than CDP-choline/Lip.

These results clearly demonstrated that CDP-choline/OX26Lip increased the therapeutic efficacy of the entrapped drug in ischemic rat models than CDP-choline/Lip, and this nanomedicine may be used for the future treatment of cerebrovascular ischemic stroke.

## Conclusion

In this study we demonstrated the improvement of CDP-choline therapeutic efficacy that has been delivered by using in OX26-conjugated liposomes with GM1 in the lipid components.

CDP-choline/OX26Lip had suitable physicochemical properties, were stable in human plasma and improved the therapeutic of CDP-choline in ischemic rat models compared to unconjugated CDP-choline/Lip. Namely, a massive reduction in the diene production was obtained when CDP-choline/OX26Lip was used (ca. 5-times less than CDP-choline/Lip). The improved efficacy of CDP-choline/OX26Lip depended on the specific binding of OX26 antibody with the transferrin receptor that is overexpressed in the BBB. This specific targeting increased the accumulation of liposomes inside the brain, promoted the brain uptake, and increased the survival rate of rats 8 days after the ischemic event (78% *vs* 96% for CDP-choline/Lip and CDP-choline/OX26Lip, respectively). These results highlighted the significant impact of OX26 antibody as targeting agents for the brain delivery and the liposomal transport across the BBB and may encourage a potential reassessment and use of CDP-choline with GM1 macromolecules for the therapy and rehabilitation of patients after of post-ischemic stroke.

## Supplementary information

Below is the link to the electronic supplementary material.

Supplementary file1 (DOCX 382 kb)

## Data Availability

The datasets generated during and/or analyzed during the current study are available from the corresponding author on reasonable request.
