# Supplementary material for: OX26-cojugated gangliosilated liposomes to improve the post-ischemic therapeutic effect of CDP-choline
Source: Drug Deliv Transl Res. 2024 Mar 13;14(10):2771–87. doi: 10.1007/s13346-024-01556-3 (PMC11384645; doi:10.1007/s13346-024-01556-3)
Supplement: Supplementary file 1 — Supplementary file1 (DOCX 382 kb) [file 13346_2024_1556_MOESM1_ESM.docx]

**OX26-cojugated gangliosilated liposomes to improve the post-ischemic therapeutic effect of CDP-choline**

Nicola d’Avanzo^1#^, Donatella Paolino^1#^, Antonella Barone^1^, Luigi Ciriolo^2^, Antonia Mancuso^1^, Maria Chiara Christiano^3^, Anna Maria Tolomeo^4,5^, Christian Celia^6,7,8^*, Xiaoyong Deng^8^*, Massimo Fresta^2^*.

^1^Department of Clinical and Experimental Medicine, University of Catanzaro “Magna Graecia”, Viale “S. Venuta”, 88100, Catanzaro, Italy.

^2^Department of Health Sciences, University of Catanzaro “Magna Graecia”, Viale “S. Venuta”, 88100, Catanzaro, Italy.

^3^Department of Medical and Surgical Sciences, University of Catanzaro “Magna Graecia”, Viale “S. Venuta”, 88100, Catanzaro, Italy.

^4^Department of Cardiac, Thoracic and Vascular Science and Public Health, University of Padua, 35128, Padua, Italy.

^5^Perdiatric Research Institute “Città della Speranza”, Corso Stati Uniti, 4, 35127, Padua, Italy.

^6^Department of Pharmacy, University of Chieti – Pescara “G. d’Annunzio”, Via dei Vestini 31, 66100, Chieti, Italy.

^7^Lithuanian University of Health Sciences, Laboratory of Drug Targets Histopathology, Institute of Cardiology, A. Mickeviciaus g. 9, LT-44307 Kaunas, Lithuania.

^8^Institute of Nanochemistry and Nanobiology, School of Environmental and Chemical Engineering, Shanghai University, Shanghai 200444, China.

^#^These authors equally contributed and a purely alphabetical order based on the surname is followed.

*Corresponding authors: these authors equally contributed and a purely alphabetical order based on the surname is followed. Celia Christian, tel. +39 0871 3554711, e-mail: c.celia@unich.it; Massimo Fresta, tel. +39 0961 3694118, e-mail: fresta@unicz.it; Xiaoyong Deng, e-mail: [xydeng@shu.edu.cn](mailto:xydeng@shu.edu.cn)

**Table S1.** Statistical analysis of Fig.4. Significance: *p < 0.05; **p < 0.01; ***p < 0.001.

| **Time points** | **PBS** | | **FBS** | |
| --- | --- | --- | --- | --- |
|  | **CDP-choline/Lip** | **CDP-choline/OX26Lip** | **CDP-choline/Lip** | **CDP-choline/OX26Lip** |
| Time_0_ *vs* time_30min_ | N.S. | N.S. | N.S. | N.S. |
| Time_0_ *vs* time_1h_ | N.S. | N.S. | N.S. | * |
| Time_0_ *vs* time_2h_ | N.S. | N.S. | * | N.S. |
| Time_0_ *vs* time_3h_ | N.S. | N.S. | * | N.S. |
| Time_0_ *vs* time_4h_ | N.S. | N.S. | N.S. | N.S. |
| Time_0_ *vs* time_6h_ | N.S. | N.S. | ** | N.S. |
| Time_0_ *vs* time_8h_ | N.S. | N.S. | * | * |
| Time_0_ *vs* time_10h_ | N.S. | N.S. | ** | ** |
| Time_0_ *vs* time_24h_ | N.S. | N.S. | *** | *** |

Footnotes: N.S.: no significant.


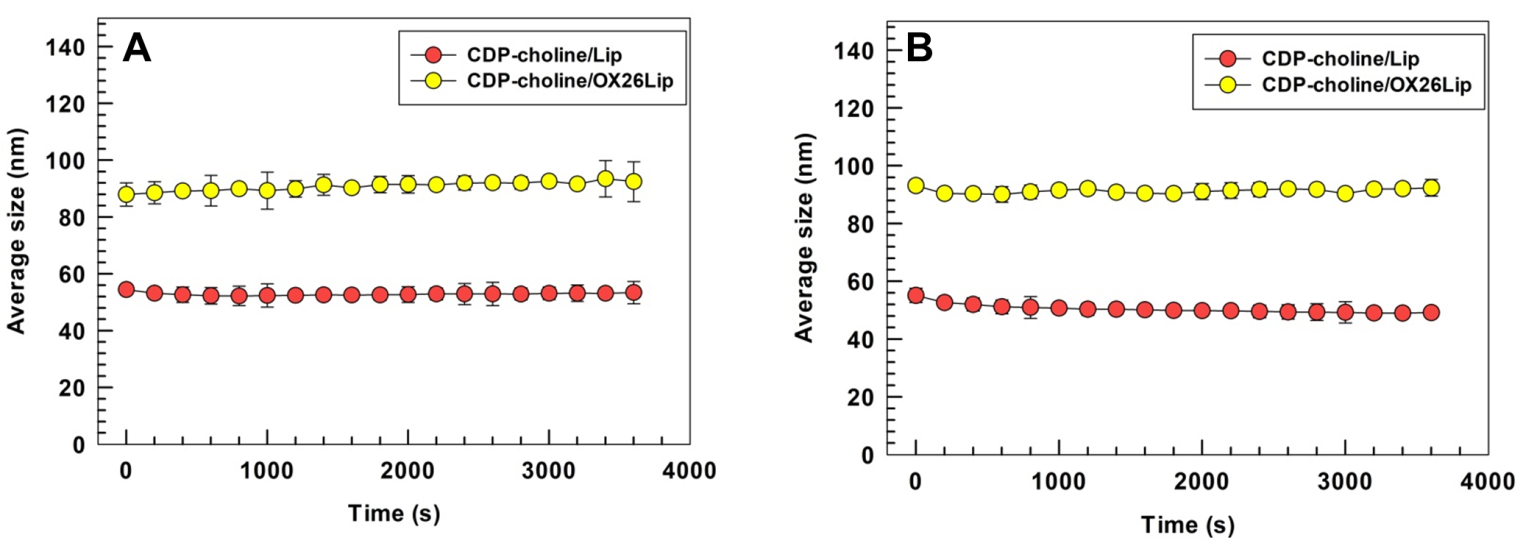


**Fig. S1.** Diameter kinetic profiles of CDP-choline/Lip and CDP-choline/OX26Lip at 25 °C (A) and 37 °C (B) during Turbiscan analysis. The mean size of liposomes is shown as a function of incubation time (0-60 min). Results are the average of three independent experiments ± S.D.
